# Phenobook: an open source software for phenotypic data collection

**DOI:** 10.1093/gigascience/giw019

**Published:** 2017-02-24

**Authors:** Juan M. Crescente, Fabio Guidobaldi, Melina Demichelis, Maria B. Formica, Marcelo Helguera, Leonardo S. Vanzetti

**Affiliations:** 1 Grupo Biotecnologia y Recursos Genéticos, EEA INTA Marcos Juárez, Ruta 12 km 3, 2580 Marcos Juárez, Argentina; 2 Consejo Nacional de Investigaciones Cientificas y Técnicas (CONICET), Argentina

**Keywords:** big data, database, data collection

## Abstract

**Background:**

Research projects often involve observation, registration, and data processing starting from information obtained in field experiments. In many cases, these tasks are carried out by several persons in different places, times, and ways, adding different levels of complexity and error in data collecting. Furthermore, data processing can be time consuming, and input errors may produce unwanted results.

**Results:**

We have developed a novel, open source software called Phenobook, an easy, flexible, and intuitive tool to organize, collect, and save experimental data for further analyses. Phenobook was conceived to collect phenotypic observations in a user-friendly, cost-effective way. It consists of a web-based software for experiment design, data input and visualization, and exportation, combined with a mobile application for remote data collecting. We provide in this article a detailed description of the developed tool.

**Conclusion:**

Phenobook is a software tool that can be easily implemented in collaborative research and development projects involving data collecting and forward analyses. Adopting Phenobook is expected to improve the involved processes by minimizing input errors, resulting in higher quality and reliability of the research outcomes.

## Background

Registration, systematization, storage, and access to large volumes of observational data are currently affecting several disciplines related to biological sciences, as many experiments base their conclusions on data obtained through observation. For example, a plant breeding program can easily contain dozens of experiment, each having hundreds of entries [1]. In other cases, ecologists collectively produce large volumes of data through diverse individual projects [2], where the variability of formats, logical structures, and sampling methods create significant challenges for downstream analyses. Cultural barriers and tradition further impede progress in the creation and adoption of data standards [3]. In agronomy, phenotype information has traditionally been captured in a free-text manner [4], possibly causing a large variation of terms and concepts used to describe comparable objects across datasets. It is not uncommon that different persons take part in data collection, so the process of writing and transcribing massive amounts of data on paper field books usually involves high costs in human resources and the risk of having poor data integrity [5]. As explained in Jones et al. [3], to address these issues scientists make use of methods like entering data in an ad hoc manner in spreadsheet-based software tools. However, these habits do not provide the tools to promote good data management practices because they lack a proper structure to adequately describe and constrain the data. As stated in Ziemann et al. [6], the spreadsheet software Microsoft Excel, when used with default settings, is known to convert gene names to dates and floating-point numbers. These errors are widespread in the scientific literature. According to a programmatic validation done by Ziemann et al. [6], approximately one-fifth of papers with supplementary Excel gene lists contain erroneous gene name conversions. A more robust way to collect data is the adoption of desktop database management systems such as Microsoft Access. The biggest limitation of these database management systems is that obtained datasets are relatively difficult to share with colleagues. There are also available tools that aim to simplify the process of data acquisition, reducing costs and enforcing data integrity, like FieldBook, developed by Rife and Poland [5], or FieldLab, developed by International Rice Research Institute [7]. These mobile applications allow users to specify the data input formats, locally save the observations in the device, and finally export results in different formats.

Other types of platforms designed for field data collecting rely on specific hardware, and they turn out to be somehow more specific (and less flexible) and high-priced than platforms previously described. Examples can be found in Berke and Baenziger [8], Liebisch et al. [9], and Busemeyer et al. [10], among others.

In this article, we describe a web-based open source software that centralizes observational records and a mobile application that can connect to the server and synchronize data, so different users can work in the same project collaboratively at the same time.

## Implementation

### Introducing Phenobook

We present Phenobook, a novel open source web-based software that handles phenotypic records, manages involved personnel, and synchronizes data with a centralized database in order to maintain data integrity and simplify data control. Phenobook is a software platform consisting of a web application that manages experiments combined with a mobile application for creating a field record of observations. It also provides up-to-date documentation, available at https://intabiotechmj.github.io/phenobook-server/.

### Users

Phenobook users are classified into three categories:

- non-administrator users;
- administrator users, who can manage other users;
- super-administrator users, who can manage other users and create users groups.

Each user belongs to a user group and can only access information created by its group. This way it is possible to use the same instance of Phenobook in different working groups. Variables are shared across the same group.

### Variables

Each Phenobook is a spreadsheet-like document. Variables are created before creating a Phenobook, so different Phenobooks can share the same variables, which makes it possible to merge results in the same report. To create variables, select the option ‘variables’ in the upper menu. Click the ‘add’ button, and insert the desired variable name, description, and field type and check if the variable is informative. The field type can be text, number, boolean, date, categorical, or photo. If a variable is selected as informative, it means that its content is known before making the observations. This will serve as a guide for the user when registering records (for example, in an experiment, cultivar and repetition variables can be both informative, with field types categorical and numerical, respectively).

### Phenobooks

New Phenobooks are created by selecting ‘Phenobooks’ – ‘add’ in the software. Each Phenobook has a name, the quantity of experimental units (rows), an optional description, and a set of variables. Once created, the Phenobook will be visible to all users within the same group of the creator. It is possible to query Phenobooks in different ways. The first one is to inspect the results of an individual Phenobook. This option shows a table with the data of the selected Phenobook. At the bottom, you can see a summary indicating creation date, last update, and completeness percentage. Extra information about the registry is provided (how the data was taken, on mobile or server, when, by whom, and a historical record with all modifications made to the record as cells with historical record are highlighted) by clicking on each cell. It is possible to fix a value (disable modification on mobile device) by clicking the ‘fix this value’ button. It is also possible to change the value to a previous one by clicking on ‘use’ this value on each historical record, and to access a variable summary by clicking on each variable name. The summary structure depends on the variable data type. Another way to query Phenobooks is the ‘Comma Separated File (CSV) Export’ option. This will create a standard CSV and download it to the user device. Finally, the ‘data report’ option makes possible to merge different Phenobooks data. Selection of which variables you want to be shown is required, and after that, the Phenobooks that will be queried must be selected. It is possible to show the results or download a CSV file. In the results table, the information for each record is available by clicking on cells, and a summary is provided when clicking on each variable name.

### Data entry from browser, spreadsheet import

Observations can be registered from the mobile application or from the server. The table shown under the option ‘Phenobooks’ – ‘Load Data Manually’ allows the user to copy and paste from/ to MS Excel, OpenOffice Calc, and similar spreadsheet software. Information is automatically saved when changed, except when the cell has a format error (i.e., alphabetic characters in a numeric variable). In this case, the cells are highlighted in red and the user is asked to correct the data.

### Mobile data entry, mobile application

Phenobook counts with a cross-platform mobile application. Android and iOS application links are available at https://github.com/INTABiotechMJ/phenobook-mobile. Moreover, advanced users can build their own versions of the application by compiling the mobile project for any supported platform by Apache Cordova, as explained in “Create your first Cordova app.” [11].

It is possible to change the Phenobook server installation URL in the mobile application

(i.e., http://yourserverip/phenobook/) by accessing the ‘settings’ menu. As the server address is changeable in the mobile application, there is no need to compile the application each time a server instance is deployed or moved to another IP address or domain.

Users can synchronize initial data by entering email and password, and pressing the ‘update’ button. Once synchronized, the user can login and see available Phenobooks.

Records are saved in the device in a local database (Web SQL), photos are encoded and saved in the database as well, and all the information is uploaded to the server as an HTTP request. Image annotations are automatically linked to data entry, so observation of anomalies can be retrospectively investigated as proposed in Burke et al. [12]. Users can trigger synchronization when connectivity to the server application is available (i.e., Internet, LAN, etc.). Through this procedure, experiment information and observations from other users are updated in the mobile device, and observations saved in local device storage are sent to the server. It can also save device GPS location (if available) and date/time when registering a variable.

Fig. 2 depicts the main interface of the Phenobook mobile application. In order to save observations in this scenario, where the Phenobook is already indicated, the user must select the variable and experimental unit and complete the datum in the ‘Value’ field.

### Data privacy and security

Both the web server and the mobile application are protected via user and password. The information is only accessible to specified users who provide valid credentials. The sessions are handled by PHP session support.

### Database specification

**Figure 1:**
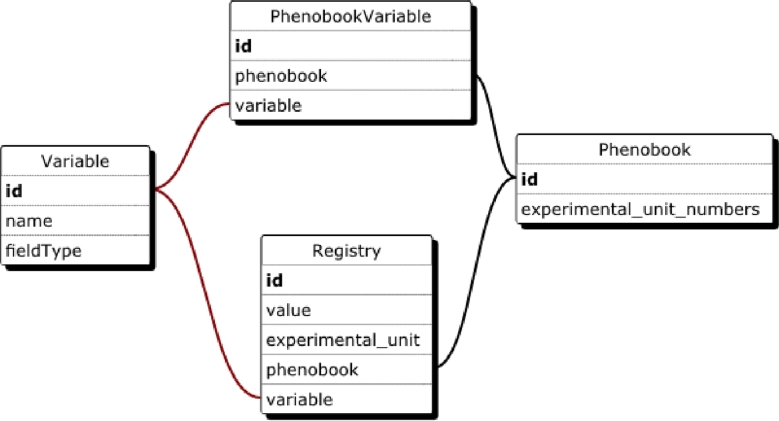
A simplified scheme of Phenobook's database.

In Fig. 1, a simplified version of the structure of the database is shown. Each registry (observation) is associated with a Phenobook and a variable and has an experimental unit number for unique identification.

**Figure 2:**
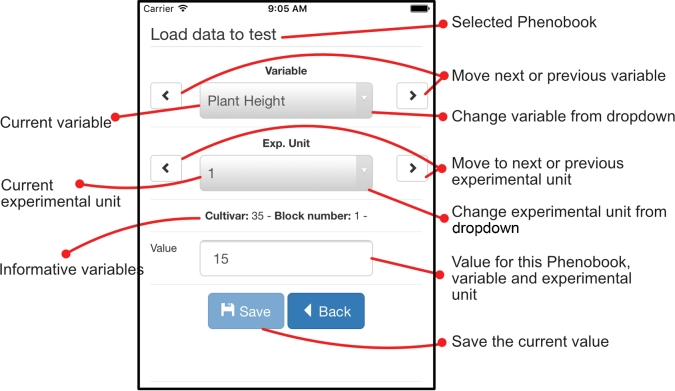
A screenshot of Phenobook mobile.

### HTTP API

It is possible to query and update the database via an HTTP Application Programming Interface (API). In order to make a request to the API, a valid username and password is required, as well as other parameters depending on the method. The API will only return data associated with the current user's group. To use this interface, parameters must be passed by GET method. Each method returns a JSON representation of the selected objects. Available methods are:

**Variables**

URL: api/export-variables.php

Parameters: user, password

Description: returns all variables available for the user's group

**Phenobooks**

URL: api/export-phenobooks.php

Parameters: user, password

Description: returns all the active Phenobooks of the user groups

**Phenobook-variables**

URL: api/export-phenobooksvariables.php

Parameters: user, password

Description: returns an associative array of Phenobooks and variables

**User groups**

URL: api/export-usergroups.php

Parameters: user, password

Description: returns all user groups

**Users**

URL: api/export-users.php

Parameters: user, password

Description: returns all users corresponding to the user's group

**Registries**

URL: api/export-registries.php

Parameters: user, password, phenobook_id, variable_id, experimental_unit

Description: returns all registries by default; possible to specify Phenobook, variable, or experimental unit as filters

**Importing**

URL: api/import-registry.php

Parameters: user, password, phenobook_id, variable_id, value

Description: saves value as a new record in specified Phenobook, variable, and experimental unit

### Deployment

It is possible to install Phenobook in a custom server. It must have PHP version 5.6 or higher, MySQL version 5.4 or higher, and Apache Server. Files in the *phenobook-server* repository must be copied to the Apache *www* folder. A MySQL database must be created in the server, and a *database.sql* file must be imported into that database; this file contains tables, structures, and one administrator user. Then, the file *files/php/config/config.php* must be updated with database details (name, username, and password). The web application is now accessible with the username *admin@yourdomain.com* and password *admin* (for security reasons, this password must be changed or this user deleted by creating another administrator user).

### Conflicting records

If the same datum (same Phenobook, variable, and experimental unit) is registered more than once, the last uploaded to the server is used by default. It is possible, however, to access all the saved data and change a value for a previous one (as explained in Phenobook Query).

### Description of tools

The web application was developed in PHP 5.6 and uses MySQL 5.4 as its database engine. The mobile application was developed in Apache Cordova, allowing multi-platform compilation. Synchronization was made via an HTTP POST request to the server, which uploads all new records (including photos) and downloads existent ones, updating the local Web SQL database.

### Comparison of Phenobook and similar tools

The idea behind Phenobook differs mainly from existing phenotype capture tools like Fieldbook [5] and Fieldlab [7] because information is stored in centralized storage thanks to a simple syncing process. This can be used by trial managers since data taken by persons involved in data capture can be easily traceable. Experiments are created only once in the server (web page) and updated in mobile devices when required. This way, data adquisition is easier to administrate and control. When the experiment is in progress, it can be seen in the server tool if an observation is taken or missing, which helps in understanding the level of completion. Furthermore, history records of each observation can be accessed, since data is not overwritten. It is feasible to know which user took which datum, when, and where. Exportation of data to well CSV is possible in all three tools. All are mobile-based applications, since data is expected to be taken in the field. Phenobook is expected to run in all major mobile phones, including other platforms besides Android (in comparison, other tools are only available for Android). This can be accomplished by compiling the source code and specifying the target platform (Android, Windows, iOS, Ubuntu, and more). This process is explained in the official Apache Cordova tutorial [11]. Image capabilities are present in all applications. Phenobook is expected to support audio recording in future releases. GPS position is saved every time a record is taken (if available), so it is possible to know where this event happened. The ability to send data to a centralized server was the main goal of this development. These characteristics allow the software to be implemented in larger work groups since it is important to have better control on users that are involved in the project, and also provide them with an easier way to share their data. This information is summarized in Table 1.

**Table 1:** Comparison between available pheno-capture tools

	Fieldbook	FieldLab	Phenobook	
Export/import CSV	yes	yes	yes	
Mobile application	yes	yes	yes	
Multi-platform	no	no	yes	
Image capabilities	yes	yes	yes	
Audio recording	yes	yes	no	
GPS position	no	no	yes	
Multiple users management	no	no	yes	
Centralized storage	no	no	yes	
Server report tool	no	no	yes	

## Conclusions

We developed Phenobook initially to manage plant breeding programs observations, although it is flexible enough to be used in wider types of experiments. The tool can be easily deployed, and it is expected to improve data quality and compatibility through exportation, simplify the processes of registering observations, and have better user control and management. The ability to trace data modifications and count with context information is also helpful when understanding how and when the data was taken.

## Availability and requirements

Project name: Phenobook

Project home page:

http://getphenobook.com

Operating system(s): platform independent

Programming language:

Server: PHP 5.4, HTML/JavaScript/CSS

Mobile application: Apache Cordova

License: Apache License 2.0

Any restrictions to use by non-academics: none

## Availability of supporting data

Further supporting data and snapshots of the code are publicly available in the GigaScience repository, GigaDB [13].

## Conflict of interest

The authors declare that they have no competing interests.

## Author contributions

J.M.C. and L.S.V. wrote the software requirement specification; J.M.C. performed the programming; L.S.V., F.G. and M.D. tested the software prototype; J.M.C. and L.S.V. drafted the manuscript; F.G., M.B.F. and, M.H. were involved in improving the manuscript. All the authors approved the final version of the manuscript.

## Supplementary Material

GIGA-D-16-00145_Original_Submission.pdf

GIGA-D-16-00145_Revision_1.pdf

Response_to_Reviewer_comments_Original_Submission.pdf

Reviewer_1_Report_Original_Submission.pdf

Reviewer_2_Report_Original_Submission.pdf
