# Supplementary material for: Phenobook: an open source software for phenotypic data collection
Source: Gigascience. 2017 Feb 24;6(4):1–5. doi: 10.1093/gigascience/giw019 (PMC5530315; doi:10.1093/gigascience/giw019)
Supplement: Response_to_Reviewer_comments_Original_Submission.pdf [file giw019_Response_to_Reviewer_comments_Original_Submission.pdf]

Dears,

we have made several corrections to our submission in order to meet the reviewer's requirements.
Below we describe all changes made and then all changes associated to each reviewer's comment.

Changes we have made to the submission:

1- Added the possibility to download variable content as a file.
2- Added an explanation in the manually update of table
3- Corrected documentation about API
4- Added pictures to documentation
5- Fix table completion indicator
6- Corrected sentence Page 2, lines 54 and 55
7- Change button label in server
8- Remove Experimental Unit unused field.
9- Minor language editing to manuscript

Changes associated to each comment.

- One issue, that was raised by myself and other reviewer, was the inability to attach external datafile to the records. This would be very useful if phenobook is aimed as a centralised data repository. For instance, you could imaging that you create a phenobook to record root architecture data. With the mobile app, you can encode the field data, and take a picture of the root system. Then, you access the data with the API and download the picture to you preferred root image analysis tool. The tools export the data to an RSML file, that you send back to the phonebook, still with the API. Like this, phenobook truly is the centralised data repository, from which you get and add data as they come.
Action:
We had added a new option to the text variables that allows user to download the content as a file. So it is possible to set the file data through the API and then download a file with its content.
We also make sure that the variable maximum size will meet ie. RSML requirements.

- In the table view, there is an issue with the completed cells information, at the bottom of the page. It always show 100% of completion, probably because the total number of cell is always 0
Action:
This has been fixed

- The manually updating the table, the data do not seems to be saved (and I did not see a save button). It seems that you have to manually double click in each cell to trigger the saving of the entry.
Action:
The table actually triggers the saving when changing any cell. An explanation has been added to the user interface.

- API does not work with credential given for the review "http://getphenobook.com/api/get-variables.php?user=test@user.com&pass=password". The request that does not need credentials (User groups) does work
Action:
This has been fixed in documentation, the actual field name for the user is email, so it should be:
http://getphenobook.com/api/export-variables.php?email=test@user.com&pass=password
Also in this particular case, it is export-variables instead of get-variables.

- I wasn't clear to me how to manage different users within the web-interface, but I guess that is because

the credential given for the review are not for an Admin right?
Action:
That's correct. We have created an Super Administrator user for you to test User Management and Group Management:
admin@user.com
password

- I would had some picture to the manual webpage, to make it easier
Action:
Pictures have been added to the manual

Manuscript comments:
- Line 55: "Each variable is represented by ???"
Action:
This has been fixed.

Reviewer #2: The Technical Note, Phenobook: An open source software for phenotypic data collection, describes a web- and mobile-based platform to collect and store plant phenotypic data collected during field (or controlled-environment) experiments. The web-based part of Phenobook is both an administrative interface that allows a user to create a Phenobook with a custom set of experimental variables, and an interface to add observations to an existing notebook. The mobile platform (both Android and iOS) allows multiple users to connect to a Phenobook to enter data, per the restrictions set up by the administrator of the book. The purpose is to standardize the collection of plant phenotypic data for an experiment, and to centralize the collection of data. An API is also available to programmatically link Phenobook to other tools. The authors have made many significant improvements to the software and the manuscript based on the previous reviewer suggestions, I only have a few minor comments.

Minor comments:
1. Page 2, lines 54 and 55: Incomplete sentence.
Action:
This issue has been fixed.

2. When adding a variable, I think the button "Existents" was meant to be "Existing" in English.
Action:
This issue has been fixed.

3. If I create a new Phenobook and add a value to the Experimental Unit text box, it does not use that value anywhere that I can see, it just uses Experimental Unit or EU.
Action:
This field has been removed since it is unused.
