# Supplementary material for: Phenobook: an open source software for phenotypic data collection
Source: Gigascience. 2017 Feb 24;6(4):1–5. doi: 10.1093/gigascience/giw019 (PMC5530315; doi:10.1093/gigascience/giw019)
Supplement: Reviewer_1_Report_Original_Submission.pdf [file giw019_Reviewer_1_Report_Original_Submission.pdf]

Reviewer Report

Title: "Phenobook: An open source software for phenotypic data collection"

Version: Original Submission **Date:** 11/30/2016

Reviewer name: Guillaume Lobet

Reviewer Comments to Author:

Note: I was reviewer 1 during the first round of reviews

The manuscript from Crescente and co-workers presents a web tool designed for the collection of phenotypic data, PhenoBook. Phonebook was created to enable research teams to encode their experimental data in a single place, to enable a better tracking and collaboration. As such, the tools relies on a centralised server to store and manage the data and a mobile app to encode them. The centralised storing is the most important feature of the tool, as it allows all the data to be stored in one place.

The authors have taken into account all the comments made by the reviewers during the first round of reviews. In my opinion, Phenobook is much more usable than it use to be, and I would like to congratulate the authors for all the development efforts. I particularly like the possibility to automatically retrieve information through an API. The copy/paste function from Excel also comes in very handy.

That said, there are still some minor technical issues (see below for details) that need to be solved before publication.

Technical issues

- One issue, that was raised by myself and other reviewer, was the inability to attach external datafile to the records. This would be very useful if phonebook is aimed as a centralised data repository. For instance, you could imaging that you create a phenobook to record root architecture data. With the mobile app, you can encode the field data, and take a picture of the root system. Then, you access the data with the API and download the picture to you preferred root image analysis tool. The tools export the data to an RSML file, that you send back to the phonebook, still with the API. Like this, phonebook truly is the centralised data repository, from which you get and add data as they come.

- In the table view, there is an issue with the completed cells information, at the bottom of the page. It always show 100% of completion, probably because the total number of cell is always 0

- The manually updating the table, the data do not seems to be saved (and I did not see a save button). It seems that you have to manually double click in each cell to trigger the saving of the entry.

- API does not work with credential given for the review "http://getphenobook.com/api/get-variables.php?user=test@user.com&pass=password". The request that does not need credentials (User groups) does work

- I wasn't clear to me how to manage different users within the web-interface, but I guess that is because the credential given for the review are not for an Admin right?

- I would had some picture to the manual webpage, to make it easier

Manuscript comments:

- Line 55: "Each variable is represented by ???"

Level of Interest

Please indicate how interesting you found the manuscript: An article of importance in its field

Quality of Written English

Please indicate the quality of language in the manuscript: Needs some language corrections before being published

Declaration of Competing Interests

Please complete a declaration of competing interests, considering the following questions:

- Have you in the past five years received reimbursements, fees, funding, or salary from an organisation that may in any way gain or lose financially from the publication of this manuscript, either now or in the future?
- Do you hold any stocks or shares in an organisation that may in any way gain or lose financially from the publication of this manuscript, either now or in the future?
- Do you hold or are you currently applying for any patents relating to the content of the manuscript?
- Have you received reimbursements, fees, funding, or salary from an organization that holds or has applied for patents relating to the content of the manuscript?
- Do you have any other financial competing interests?
- Do you have any non-financial competing interests in relation to this paper?

If you can answer no to all of the above, write 'I declare that I have no competing interests' below. If your reply is yes to any, please give details below.

I declare that I have no competing interests

I agree to the open peer review policy of the journal. I understand that my name will be included on my report to the authors and, if the manuscript is accepted for publication, my named report including any attachments I upload will be posted on the website along with the authors' responses. I agree for my report to be made available under an Open Access Creative Commons CC-BY license (http://creativecommons.org/licenses/by/4.0/). I understand that any comments which I do not wish to be included in my named report can be included as confidential comments to the editors, which will not be published.

I agree to the open peer review policy of the journal
