# Supplementary material for: Phenobook: an open source software for phenotypic data collection
Source: Gigascience. 2017 Feb 24;6(4):1–5. doi: 10.1093/gigascience/giw019 (PMC5530315; doi:10.1093/gigascience/giw019)
Supplement: Reviewer_2_Report_Original_Submission.pdf [file giw019_Reviewer_2_Report_Original_Submission.pdf]

Reviewer Report

Title: "Phenobook: An open source software for phenotypic data collection"

Version: Original Submission **Date:** 12/6/2016

Reviewer name: Noah Fahlgren

Reviewer Comments to Author:

The Technical Note, Phenobook: An open source software for phenotypic data collection, describes a web- and mobile-based platform to collect and store plant phenotypic data collected during field (or controlled-environment) experiments. The web-based part of Phenobook is both an administrative interface that allows a user to create a Phenobook with a custom set of experimental variables, and an interface to add observations to an existing notebook. The mobile platform (both Android and iOS) allows multiple users to connect to a Phenobook to enter data, per the restrictions set up by the administrator of the book. The purpose is to standardize the collection of plant phenotypic data for an experiment, and to centralize the collection of data. An API is also available to programmatically link Phenobook to other tools.

The authors have made many significant improvements to the software and the manuscript based on the previous reviewer suggestions, I only have a few minor comments.

Minor comments:

1. Page 2, lines 54 and 55: Incomplete sentence.

2. When adding a variable, I think the button "Existents" was meant to be "Existing" in English.

3. If I create a new Phenobook and add a value to the Experimental Unit text box, it does not use that value anywhere that I can see, it just uses Experimental Unit or EU.

Level of Interest

Please indicate how interesting you found the manuscript: An article of importance in its field

Quality of Written English

Please indicate the quality of language in the manuscript: Acceptable

Declaration of Competing Interests

Please complete a declaration of competing interests, considering the following questions:

- Have you in the past five years received reimbursements, fees, funding, or salary from an organisation that may in any way gain or lose financially from the publication of this manuscript, either now or in the future?
- Do you hold any stocks or shares in an organisation that may in any way gain or lose financially from the publication of this manuscript, either now or in the future?
- Do you hold or are you currently applying for any patents relating to the content of the manuscript?
- Have you received reimbursements, fees, funding, or salary from an organization that holds or has applied for patents relating to the content of the manuscript?
- Do you have any other financial competing interests?
- Do you have any non-financial competing interests in relation to this paper?

If you can answer no to all of the above, write 'I declare that I have no competing interests' below. If your reply is yes to any, please give details below.

I declare that I have no competing interests

I agree to the open peer review policy of the journal. I understand that my name will be included on my report to the authors and, if the manuscript is accepted for publication, my named report including any attachments I upload will be posted on the website along with the authors' responses. I agree for my report to be made available under an Open Access Creative Commons CC-BY license (http://creativecommons.org/licenses/by/4.0/). I understand that any comments which I do not wish to be included in my named report can be included as confidential comments to the editors, which will not be published.

I agree to the open peer review policy of the journal
